# Supplementary material for: In vivo characterisation of fluorescent proteins in budding yeast
Source: Sci Rep. 2019 Feb 19;9:2234. doi: 10.1038/s41598-019-38913-z (PMC6381139; doi:10.1038/s41598-019-38913-z)
Supplement: Supplementary file 1 — Supplementary info [file 41598_2019_38913_MOESM1_ESM.pdf]

In vivo characterisation of fluorescent proteins in budding yeast.

Dennis Botman, Daan Hugo de Groot, Phillipp Schmidt, Joachim Goedhart, Bas Teusink

Creation of constructs

sYFP2-T2A-mTq2, tagRFPT-T2A-mTq2, tdTomato-T2A-mTq2, tagRFP-T2A-mTq2, mCherry-T2A-mTq2, mCherry-T2A-eGFP, mCherry-T2A-sGFP2, YPET-T2A-mTq2, Citrine-T2A-mTq2, mNeongreen-T2A-mTq2, mKOκ-T2A-mTq2, Clover-T2A-mTq2, mRuby2-T2A-mTq2, mScarlet-T2A-mTq2, mScarletI-T2A-mTq2 and mKate2-T2A-mTq2 were based on mKO2-T2A-mTq2 (addgene plasmid #98838). The plasmids mVenus-mTq2, eCFP, mTq2 and mTFP in a clontech style C1 mammalian expression vector and mCherry in a clontech style N1 mammalian expression vector were made by restriction enzyme based cloning.

pFA6a-link-yoSuperfolderGFP-CaURA3 (Addgene plasmid #44873), pFA6a-link-yomCherry-CaURA3 (Addgene plasmid #44876), pFA6a-link-yoTagRFP-T-CaURA3 (Addgene plasmid #44877), pFA6a-link-yoEGFP-CaURA3 (Addgene plasmid #44872) were a gift from Wendell Lim & Kurt Thorn. pKT90 (pFA6a–link–yEVenus–SpHIS5, Addgene plasmid #8714) and pKT174 (pFA6a–link–yECFP–CaURA3, Addgene plasmid #8720, named yoeCFP in this study) were a gift from Kurt Thorn.

The yeast expression vector pDRF1-GW (a gift from Wolf Frommer & Dominique Loque, Addgene plasmid #36026) with the NheI and NotI restriction sites was created using the GateWay Kit (Thermo Fisher Scientific, Waltham, MA, USA).

sYFP2-T2A-mTq2, tagRFPT-T2A-mTq2, tdTomato-T2A-mTq2, tagRFP-T2A-mTq2, mCherry-T2A-mTq2, YPET-T2A-mTq2, Citrine-T2A-mTq2, mNeongreen-T2A-mTq2, mKO2-T2A-mTq2, mKOκ-T2A-mTq2, Clover-T2A-mTq2, mRuby2-T2A-mTq2, mScarlet-T2A-mTq2, mScarletI-T2A-mTq2 and mKate2-T2A-mTq2 were digested using NheI and NotI (New England Biolabs, Ipswich, Massachusetts, USA) and ligated into pDRF1 diggested with the same enzymes using T4 ligase (New England Biolabs) which created pDRF1 containing sYFP2, tagRFP, tagRFPT, mCherry, Citrine, mNeongreen, YPET, mKO2, mKOκ, Clover, mRuby2, tdTomato, mKate2, mScarlet and mScarletI fused with T2A-mTq2 in pDRF1.

mVenus-T2A-mTq2 was created by digesting mVenus-mTq2 C1 with NheI and Kpn2I. Next, the digested fragment was ligated using T4 ligase in mCherry-T2A-mTq2 digested with the same enzymes, replacing mCherry with mVenus.

sYFP2-T2A-mCherry C1 was created by digesting mCherry N1 with NotI and BamHI (New England Biolabs). Next, the digested fragment was ligated using T4 ligase in sYFP2-T2A-mTq2 C1 digested with the same enymes, replacing mTq2 for mCherry.

To create yosfGFP-T2A-mCherry, eGFP-T2A-mCherry, yoeGFP-T2A-mCherry and sGFP2-T2A-mCherry, a PCR using KOD polymerase (Merck-Millipore, Burlington, Massachusetts, USA) was performed according to table S1. The products and FP-T2A-mTq2 pDRF1 were digested with NheI and Kpn2I (Thermo Fisher Scientific, Waltham, Massachusetts, USA) and the products were ligated into the plasmid, replacing the FP N-terminally of T2A-mTq2 with yosfGFP, eGFP, yoeGFP and sGFP2. Next, mTq2 was cut out of eGFP-T2A-mTq2, mNeongreen-T2A-mTq2, sGFP2-T2A-mTq2, yosfGFP-T2A-mTq2 and yoeGFP-T2A-mTq2 in pDRF1 using Kpn2I and NotI and replaced by mCherry digested from sYFP2-T2A-mCherry using the same enzymes.

To create yoeCFP-T2A-mCherry, yotagRFPT-T2A-mTq2, yomCherry-T2A-mTq2, yeVenus-T2A-mTq2 and mTq2-T2A-mCherry, PCRs with KOD polymerase were performed according to table S1, the products were digested with NheI and Kpn2I and ligated with T4 ligase into a T2A-mCherry or T2A-mTq2 pDRF1 vector in which the FP N-terminally of T2A was removed by digestion with the same enzymes. This generated yoeCFP-T2A-mCherry, yotagRFPT-T2A-mTq2, yomCherry-T2A-mTq2, yeVenus-T2A-mTq2 and mTq2-T2A-mCherry in pDRF1.

Lastly, eCFP-C1 and mTFP-C1 were digested with NheI and Kpn2I and ligated into mNeongreen-T2A-mCherry in pDRF1 digested with NheI and Kpn2I which replaced mNeongreen with either eCFP or mTFP.

yFPs

tdTomato, mScarletI, and mYPET (YPET A206K, F208S, E231L, N234D) were codon-optimized and synthesised (Baseclear B.V., Leiden, The Netherlands), generating ytdTomato, ymScarletI and ymYPET. These constructs were digested with NheI and Kpn2I and ligated using T4 ligase into either T2A-mTq2 or T2A-mCherry in which the FP N-terminally of T2A was removed by digestion with the same enzymes. This generated ytdTomato-T2A-mTq2, ymScarletI-T2A-mTq2, ymNeongreen-T2A-mTq2, ymNeongreen-T2A-mCherry and ymYPET-T2A-mTq2 in pDRF1.

Msn2-ymNeongreen and ymTq2Δ9 pUC19 plasmids were codon-optimized and synthesised (Baseclear). A PCR was performed using these constructs according to table S1. Next, the products were digested using NheI and Kpn2I and ligated using T4 ligase into T2A-mTq2 and T2A-mCherry pDRF1 plasmids in which the FP N-terminally of T2A was removed by digestion with the same enzymes, which generated ymTq2-T2A-mCherry, ymNeongreen-T2A-mTq2 and ymNeongreen-T2A-mCherry.

pDRF1 plasmids containing the single yFPs were generated by performing a PCR according to table S1 on Msn2-ymNeongreen, ymTq2Δ9, ymYPET, ytdTomato, ymScarletI in pUC19 plasmids which added a stopcodon at the C-termini. Subsequently, the PCR products were digested with NheI and NotI and ligated with T4 ligase in an empty pDRF1 vector digested with NheI and NotI which generated ymYPET, ymTq2, ymScarletI, ytdTomato and ymNeongreen in pDRF1.

CytERM-ymVenus was created by a mutagenesis PCR according to table S1. Afterwards, pDRF1 containing ymVenus-T2A-mTq2 was constructed by performing a PCR on CytERM-ymVenus according to table S1. Next, the product was digested using NheI and Kpn2I and ligated into a T2A-mTq2 pDRF1 vector in which the FP N-terminally of T2A was removed by digestion with the same enzymes.

pFA6a-yFP-CaURA3 plasmids containing the yFPs were generated by performing a PCR according to table S1. Next, the products were digested using PacI and AscI (New England Biolabs) and ligated with T4 ligase into the plasmid pFA6a-link-yomCherry-CaURA3 also digested with PacI and AscI to replace yomCherry with the yFP, which generated pFA6a-yFP-CaURA3 plasmids.

pFA6a-link-ymNeongreen-SpHis5 was generated by performing a PCR on Msn2-ymNeongreen pUC19 according to table S1. Next, the product was digested using PacI and AscI and ligated into pFA6a-link-yomKate2-SpHis5 also digested with PacI and AscI (New England Biolabs), replacing yomKate2 with ymNeongreen.

CytERM constructs

CytERM-dTomato (addgene plasmid #98834) and CytERM-mTq2 (addgene plasmid #98833) were digested using NheI and NotI and ligated into an empty pDRF1 vector digested with the same enzymes which generated CytERM-dTomato and CytERM-mTq2 in pDRF1. CytERM-yeVenus, CytERM-ymNeongreen, CytERM-ytdTomato, CytERM-ymScarletI, CytERM-ymTq2 and CytERM-ymYPET pDRF1 were created by performing a PCR according to table S1. Afterwards, products were digested using XmaI (New England Biolabs) and NotI and ligated with T4 ligase into a CytERM pDRF1 plasmid in which the FP C-terminally of CytERM was removed by XmaI and NotI which generated the CytERM-yFPs.

Table S1. Constructs generated by PCR in this study.

Construct	Template used for PCR	FW primer	RV primer
yosfGFP-T2A-mTq2 pDRF1	pFA6a-link-yoSuperfolderGFP-CaURA3 (Addgene plasmid # 44873)	ATGCTAGCCACCATGTCTAAAGGCGAGGAATT	TCTCCGGATTTGTACAATTCGTCCATTCC
eGFP-T2A-mTq2 pDRF1	mCherry-T2A-eGFP C1	ATGCTAGCCACCATGGTGAGCAAGGGC	TATCCGGACTTGTACAGCTCGTCCA
yoeGFP-T2A-mTq2 pDRF1	pFA6a-link-yoEGFP-CaURA3 (Addgene plasmid # 44872)	ATGCTAGCCACCATGTCAAAAGGCGAGGAAC	TCTCCGGACTTGTATAATTCATCCATGCCC
sGFP2-T2A-mTq2 pDRF1	mCherry-T2A-sGFP2 C1	ATGCTAGCCACCATGGTGAGCAAGGGC	TATCCGGACTTGTACAGCTCGTCCA
yeVenus-T2A-mTq2 pDRF1	pFA6a–link–yEVenus–SpHIS5 (Addgene plasmid # 8714)	ATGCTAGCCACCATGTCTAAAGGTGAAGAATTATTCAC	TCTCCGGATTTGTACAATTCATCCATACCAT

yeCFP-T2A-mCherry pDRF1	pFA6a–link–yECFP–CaURA3 (Addgene plasmid #8720)	ATGCTAGCCACCATGTCTAAAGGTGAAGAATTATTCAC	TCTCCGGATTTGTACAATTCATCCATACCAT
mTq2-T2A-mCherry pDRF1	mTq2 C1	CTGCTAGCGCTACCGG	TATCCGGACTTGTACAGCTCGTCCA
yotagRFPT-T2A-mTq2 pDRF1	pFA6a-link-yoTagRFP-T-CaURA3 (Addgene plasmid #44877)	ATGCTAGCCACCATGGTATCTAAAGGTGAAGAGTTG	TCTCCGGACTTATACAATTCATCCATACCATTCAG
yomCherry-T2A-mTq2 pDRF1	pFA6a-link-yomCherry-CaURA3 (Addgene plasmid #44876)	ATGCTAGCCACCATGGTTAGCAAAGGCGAG	TATCCGGACTTGTACAGTTCATCCATACCA
ymNeongreen-T2A-mCherry pDRF1	Msn2-ymNeongreen pUC19	ATGCTAGCACTAGTAAGCTTTTAATTAAATGGTCTCTAAGGGTGAAGA	ATGCGGCCGCCTCGAGGTCGACGGCGCGCCTCCGGACTTGTACAATTCGTCCATACC
ymNeongreen-T2A-mTq2 pDRF1	Msn2-ymNeongreen pUC19	ATGCTAGCACTAGTAAGCTTTTAATTAAATGGTCTCTAAGGGTGAAGA	ATGCGGCCGCCTCGAGGTCGACGGCGCGCCTCCGGACTTGTACAATTCGTCCATACC
ymTq2-T2A-mCherry	ymTq2Δ9 pUC19	ATGCTAGCACTAGTAAGCTTTTAATTAAATGGTTAGTAAAGGTGAAGAA	ATTCCGGATTTATACAATTCATCCATACCTAAAGTGATCCCAGCAGCAGT
CytERM-yeVenus pDRF1	pFA6a–link–yEVenus–SpHIS5 (Plasmid #8714)	ATCCCGGGATCCACCGGTCGCCACCATGTCTAAAGGTGAAGAATTATTCAC	ATGCGGCCGCTTATTTGTACAATTCATCCATACCAT
CytERM-ymVenus pDRF1	CytERM-yeVenus pDRF1	TATCCTATCAATCTAAATTATCCAAAGATCC	GGATCTTTGGATAATTTAGATTGATAGGATA
ymVenus-T2A-mTq2 pDRF1	CytERM-ymVenus pDRF1	ATGCTAGCACTAGTAAGCTTTTAATTAAATGTCTAAAGGTGAAGAATTATTCAC	ATGCGGCCGCCTCGAGGTCGACGGCGCGCCTCCGGATTTGTACAATTCATCCATACCATG
CytERM-yeVenus pDRF1	pFA6a–link–yEVenus–SpHIS5 (Addgene plasmid # 8714)	ATCCCGGGATCCACCGGTCGCCACCATGTCTAAAGGTGAAGAATTATTCAC	ATGCGGCCGCTTATTTGTACAATTCATCCATACCAT
ymYPET pDRF1	ymYPET pUC19	ATGCTAGCACTAGTAAGCTTTTAATTAA	ATTCCGGAGGCGCGCCGTCGACCTCGAGGCGGCCGCTTATTTATACAATTCATCCATACCTAAAGTAATACC
ymTq2 pDRF1	ymTq2Δ9 pUC19	ATGCTAGCACTAGTAAGCTTTTAATTAAATGGTTAGTAAAGGTGAAGAA	ATGCGGCCGCTTATTTATACAATTCATCCATACCTAAAGTGATCCCAGCAGCAGT
ymScarletI pDRF1	ymScarletI pUC19	ATGCTAGCACTAGTAAGCTTTTAATTAA	ATTCCGGAGGCGCGCCGTCGACCTCGAGGCGGCCGCTTATTTATACAATTCATCCATTCCTCCG
ytdTomato pDRF1	ytdTomato pUC19	ATGCTAGCACTAGTAAGCTTTTAATTAA	ATTCCGGAGGCGCGCCGTCGACCTCGAGGCGGCCGCTTATTTATATAATTCATCCATACCGTATAAAAAC
ymNeongreen pDRF1	Msn2-ymNeongreen pUC19	ATGCTAGCACTAGTAAGCTTTTAATTAAATGGTCTCTAAGGGTGAAGA	ATGCGGCCGCCTCGAGGTCGACGGCGCGCCTCCGGACTTGTACAATTCGTCCATACC
pFA6a-ymYPET-CaURA3	ymYPET pUC19	ATGCTAGCACTAGTAAGCTTTTAATTAA	ATTCCGGAGGCGCGCCGTCGACCTCGAGGCGGCCGCTTATTTATACAATTCATCCATACCTAAAGTAATACC
pFA6a-ymTq2-CaURA3	ymTq2Δ9 pUC19	ATGCTAGCACTAGTAAGCTTTTAATTAAATGGTTAGTAAAGGTGAAGAA	ATTCCGGAGGCGCGCCGTCGACCTCGAGGCGGCCGCTTATTTATACAATTCATCCATACCTAAAG
pFA6a-ymScarletI-CaURA3	ymScarletI pUC19	ATGCTAGCACTAGTAAGCTTTTAATTAA	ATTCCGGAGGCGCGCCGTCGACCTCGAGGCGGCCGCTTATTTATACAATTCATCCATTCCTCCG
pFA6a-ytdTomato-CaURA3	ytdTomato pUC19	ATGCTAGCACTAGTAAGCTTTTAATTAA	ATTCCGGAGGCGCGCCGTCGACCTCGAGGCGGCCGCTTATTTATATAATTCATCCATACCGTATAAAAAC
pFA6a-ymNeongreen-CaURA3	Msn2-ymNeongreen pUC19	ATGCTAGCACTAGTAAGCTTTTAATTAAATGGTCTCTAAGGGTGAAGA	ATTCCGGAGGCGCGCCGTCGACCTCGAGGCGGCCGCTTACTTGTACAATTCGTCCATACCC
pFA6a-ymVenus-CaURA3	CytERM-yeVenus pDRF1	ATGCTAGCACTAGTAAGCTTTTAATTAAATGTCTAAAGGTGAAGAATTATTCAC	ATTCCGGAGGCGCGCCGTCGACCTCGAGGCGGCCGCTTATTTGTACAATTCATCCATACCATG
pFA6a-link-ymNeongreen SpHis5	Msn2-ymNeongreen pUC19	GTTTAATTAACATGGTCTCTAAGGG	ATGGCGCGCCTTACTTGTACAATTCGTCCATAC
CytERM–ymNeongreen pDRF1	ymNeongreen pDRF1	ATCCCGGGATCCACCGGTCGCCACCATGGTCTCTAAGGGTGAAGA	ATTCCGGAGGCGCGCCGTCGACCTCGAGGCGGCCGCTTACTTGTACAATTCGTCCATACCC
CytERM–ytdTomato pDRF1	ytdTomato pDRF1	ATCCCGGGATCCACCGGTCGCCACCATGGTTAGTAAAGGTGAGGAAG	ATTCCGGAGGCGCGCCGTCGACCTCGAGGCGGCCGCTTATTTATATAATTCATCCATACCGTATAAAAAC

CytERM–ymScarletI pDRF1	ymScarletI pDRF1	ATCCCGGGATCCACCGGTCGCCACCATGGTCTCCAAGGGCG	ATTCCGGAGGCGCGCCGTCGACCTCGAGGCGGCCGCTTATTTATACAATTCATCCATACCTAAAGTAATACC
CytERM–ymTq2 pDRF1	ymTq2 pDRF1	ATCCCGGGATCCACCGGTCGCCACCATGGTTAGTAAAGGTGAAGAATTG	ATGCGGCCGCTTATTTATACAATTCATCCATACCTAAAGTGATCCCAGCAGCAGT
CytERM–ymYPET pDRF1	ymYPET pDRF1	ATCCCGGGATCCACCGGTCGCCACCATGGTCTCCAAGGGCG	ATTCCGGAGGCGCGCCGTCGACCTCGAGGCGGCCGCTTATTTATACAATTCATCCATACCTAAAGTAATACC

*Table S1. Extended in vivo properties of the yFPs. [a]Spectral color class. [b]Excitation maximum. [c]Emission maximum. [d]Brightness in budding yeast normalized to either mCherry (for CFPs and GFPs) or mTq2 (for YFPs and RFPs), determined by the mean of the mean brightness of 3 days. [e]Fluorescence fractions remaining at the last timeframe, normalized to the first frame. [f]T-half times in seconds obtained via a one-phase or two-phase exponential decay fit. Cell-to-cell variation, depicted by the coefficient of variation between the mean brightness of the 3 days. [h]Fluorescence lifetime in nanoseconds determined by frequency domain fluorescence lifetime imaging. [i]Monomerism depicted by the mean amount of OSER structures per cell. [j]pH value giving 50% decrease in fluorescence. [k]Hill coefficient of the pH stability. [l]pH value giving an absolute decrease of 50% in fluorescence. Abbreviations: SD; standard deviation. *eCFP obtained from Hui-wang AI et al 2006. mkokappa from Tsutsui et al 2008. mScarlet and mScarletI from Bindels et al 2006. sGFP2 from Kremers et al 2007. sYFP2 from Kremers et al 2006. Others from Cranfill et al as these are all determined the same way.*

Construct	FP analyzed (FP of interest)	Color[a]	Exc. max[b]	Em. max[c]	*In vivo* brightness[d]	day-to-day variation[g]	*in vitro* brightness*	brightness to mTq2 or mcherry	*in vivo-in vitro* brightness difference	Photostability[e]	SD photostability	T_{half} 1(sec)[f]	T_{half} 2 (sec)[f]	Life-time[h]	Monomerism ± SD[i]	*in vivo* pKa[j]	Hill coeff.[k]	$pH_{50\%}$[l]	pKa literature	pKa *in vivo* - pKa literature	Photochromism for RFP excitation	Photochromism for GFP excitation	Photochromism for CFP excitation	Photochromism for YFP excitation
**eCFP-T2A-mCherry**	eCFP	CFP			1.43	0.23	13.00	0.51	0.92	0.72	0.08	19.084	0.110			4.17	0.52	3.99	4.7	-0.53	0.88	-46.59	-20.89	-61.26
**eGFP-T2A-mCherry**	eGFP	GFP			1.98	0.13	37.52	1.47	0.50	0.91	0.03	3075.187	27.289			5.96	0.67	5.95	6.1	-0.14	-105.36	-810.77	-18670.09	
**mCherry-T2A-mTq2**	mCherry	RFP			0.51	0.16	25.50	0.89	-0.39	0.92	0.02	67.690	2.888			3.45	0.91	3.45	3.8	-0.35	-19.51	-35.75	-57.61	-10.08
**Citrine-T2A-mTq2**	Citrine	YFP			1.32	0.09	81.90	2.87	-1.55	0.71	0.02	27.108	0.124			5.62	0.58	5.58	5.4	0.22	1.22		-22.52	-4.14
**Clover-T2A-mTq2**	Clover	YFP			0.58	0.29	92.40	3.24	-2.66	0.64	0.03	120.526	0.923			6.47	0.75	6.38	5.9	0.57	-83.46	-61.49	-2.30	-3.50
**mKate2-T2A-mTq2**	mKate2	RFP			1.36	0.61	24.15	0.85	0.51	0.89	0.03	61.613	0.555			4.76	0.51	4.76	5.5	-0.74	-1.23	-14.57	-13.97	-14.87
**mko2-T2A-mTq2**	mKo2	YFP			0.10	0.56	74.55	2.61	-2.51	0.78	0.03	29.647	0.205			5.51	0.54	5.51	5.5	0.01			191.90	-3.18
**mkok-T2A-mTq2**	mkok	YFP			0.17	0.43	64.05	2.25	-2.08	0.71	0.02	4.329	0.012			4.50	0.62	4.50	4.2	0.30			6135.63	-4.68
**mNeongreen-T2A-mCherry**	mNeongreen	GFP			3.39	0.01	90.40	3.55	-0.15	0.99	0.03	0.190				5.44	0.92	5.44	5.4	0.04	-0.37	-0.02	-0.33	-0.19
**mNeongreen-T2A-mTq2**	mNeongreen	YFP			2.00	0.05	90.40	3.17	-1.17	0.96	0.01	0.110	0.002			5.44	0.92	5.44	5.4	0.04				
**mRuby2-T2A-mTq2**	mRuby2	RFP			0.34	0.59	39.59	1.39	-1.04	0.68	0.01	70.427	0.823			4.77	0.45	4.77	4.4	0.37	-4.20	497.15	485.59	386.32
**mScarletI-T2A-mTq2**	mScarletI	RFP			1.72	0.16	57.00	2.00	-0.27	0.75	0.02	108.508				3.98	0.42	4.85	5.4	-1.42	-63.74	-79.36	-88.46	-61.48
**mScarlet-T2A-mTq2**	mScarlet	RFP			0.66	0.10	71.00	2.49	-1.83	0.64	0.08	4642.647				4.85	0.38	3.98	5.3	-0.45	-10.18	6.66	6.43	7.75
**mTFP-T2A-mCherry**	mTFP	CFP			0.89	0.06	45.05	1.77	-0.88	0.95	0.02	461.790	1.384			4.92	0.54	4.92	4.3	0.62	2.06	0.93	-0.17	2.63
**mTq2-T2A-mcherry**	mTq2	CFP			1.82	0.12	28.52	1.12	0.70	0.92	0.02	2559.628	9.790			4.51	0.62	4.51	3.6	0.91	15.24	-21.99	-0.37	-0.18
**mVenus-T2A-mTq2**	mVenus	YFP			1.15	0.22	85.09	2.98	-1.83	0.47	0.06	41.481	0.103			5.45	0.65	5.45	5.4	0.05	-7.36		-12.65	-10.56
**sGFP2-T2A-mCherry**	sGFP2	GFP			1.85	0.13	32.20	1.26	0.58	0.86	0.03	27.097	0.124			6.14	0.58	6.08	5.9	0.24	85.88	-435.71	-1009.19	
**sYFP2-T2A-mTq2**	sYFP2	YFP			1.26	0.33	68.68	2.41	-1.14	0.34	0.02	19.753	0.136			6.14	0.72	5.98	6.0	0.14	-1.64		-45.23	-40.98
**tagRFP-T2A-mTq2**	tagRFP	RFP			0.21	0.66	42.90	1.50	-1.30	0.44	0.03	71.799	1.060			2.99	1.13	2.99	3.0	-0.01	-4.52	561.57	731.40	21863.39
**tagRFPT-T2A-mTq2**	tagRFPT	RFP			0.19	0.11	33.90	1.19	-1.00	0.84	0.04	43.160	0.114			4.27	0.52	4.27	4.3	-0.03	-1.48	14.44	49.11	4.47
**tdTomato-T2A-mTq2**	tdTomato	RFP			1.44	0.14	50.60	1.77	-0.34	0.92	0.03	74.324	2.126			4.57	0.98	4.57	4.5	0.07	-1.67	-4.56	3.63	-4.25
**yeVenus-T2A-mTq2**	yeVenus	YFP			1.39	0.25	81.90	2.87	-1.48	0.53	0.05	24.623	0.085		0.51 ± 0.89	5.51	0.60	5.51	5.6	-0.09	-5.95		-54.41	-6.21
**ymNeongreen-T2A-mCherry**	ymNeongreen	GFP	506	517	4.16	0.03	90.40	3.55	0.62	0.98	0.01	0.191	0.001	3.2	0.15 ± 0.60	5.42	1.08	5.42	5.4	0.02				
**ymNeongreen-T2A-mTq2**	ymNeongreen	YFP	506	517	2.48	0.05	90.40	3.17	-0.69	0.95	0.02	5752.259	33.812	3.2	0.15 ± 0.60	5.42	1.08	5.42	5.4	0.02				
**ymScarletI-T2A-mTq2**	ymScarletI	RFP	568	591	1.02	0.12	57.00	2.00	-0.98	0.74	0.07	117.802		n.d.	0.15 ± 0.42	4.26	0.44	4.26	5.4	-1.14				
**ymTq2-T2A-mCherry**	ymTq2	CFP	439	475	2.24	0.30	28.52	1.12	1.12	0.94	0.03	489.857		4	0.20 ± 0.42	4.48	0.64	4.48	3.6	0.88				
**ymVenus-T2A-mTq2**	ymVenus	YFP	515	527	1.90	0.14	85.09	2.98	-1.08	0.62	0.03	31.913	0.403	2.9	0.49 ± 0.81	5.50	0.62	5.50	5.4	0.10				
**ymYPET-T2A-mTq2**	ymYPET	YFP	516	526	1.72	0.13				0.64	0.04	46.272	0.139	3	0.12 ± 0.40	4.89	0.91	4.89						
**yoeCFP-T2A-mCherry**	yoeCFP	CFP			0.42	0.07				0.88	0.02	40.797	0.099			4.76	3.05	4.74						
**yoeGFP-T2A-mCherry**	yoeGFP	GFP			1.65	0.10	45.88	1.80	-0.14	0.90	0.02	72.452	1.222			6.04	0.65	6.04	6.1	-0.06	-1.21	-3.35	-219.75	
**yomCherry-T2A-mTq2**	yomCherry	RFP			0.53	0.16	25.50	0.89	-0.36	0.91	0.03	116.125	0.715			3.43	0.84	3.43	3.8	-0.37	-1.09	-2.44	-3.38	-0.78
**yosfGFP-T2A-mCherry**	yosfGFP	GFP			1.94	0.11	38.16	1.50	0.44	0.81	0.02	87.243	0.733			6.11	0.73	6.09	5.8	0.31	-19.11	-50.81	-106.70	
**yotagRFPT-T2A-mTq2**	yotagRFPT	RFP			1.07	0.06	33.90	1.19	-0.12	0.94	0.01	804.022				4.65	0.87	4.65	4.3	0.35	0.00	0.04	0.02	0.03
**YPET-T2A-mTq2**	YPET	YFP			3.91	0.19	100.30	3.52	0.40	0.74	0.03	41.481	0.103			4.81	0.74	4.81	5.5	-0.69	-178.11		-375.44	-4.95
**ytdtomato-T2A-mTq2**	ytdTomato	RFP	552	580	1.01	0.07	50.60	1.77	-0.76	0.95	0.01	1459.872		n.d.	0.30 ± 0.67	4.76	1.14	4.76	4.5	0.26				

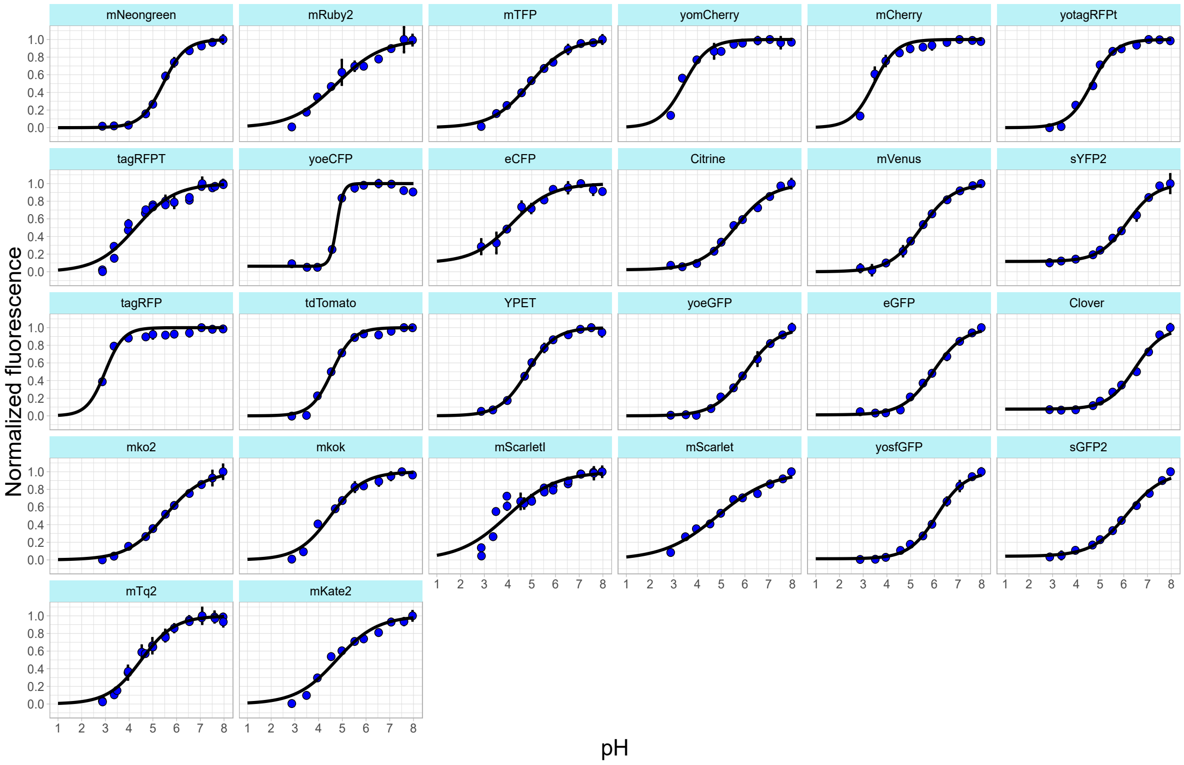

Figure S1. pH sensitivity of all characterised FPs. Yeast cells were incubated for 2 hours in a citric-acid/Na_2HPO_4 buffer with 2 mM DNP and fluorescence was measured using a fluorescent plate reader. Per FP, at least 3 technical replicates were measured. Afterwards, fluorescence was normalized to the pH giving the highest fluorescence and a hill fit was performed to determine the hill coefficient and pKa value. Dots represent the mean of at least 3 replicates, line indicates fitted pH response curve, error bars indicate SD.

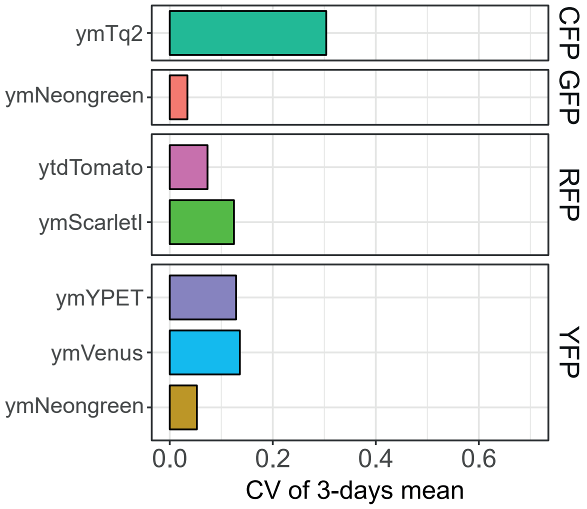

Figure S2. Day-to-day variation of yFPs depicted by the coefficient of variation (CV) of the mean brightness of 3 days.

Model of photochromicity

1 Model

Bleaching of Fluorescent Proteins (FPs) is often modelled by a system of linear differential equations[Dickson et al., 1997, Dean et al., 2011, Morisaki and McNally, 2014, Sinnecker et al., 2005]. These models incorporate different states with transition rates that describe blinking (fast switching between a fluorescent state and a non-fluorescent state), bleaching and irreversible bleaching behaviour. Since we are only interested in photochromicity and bleaching, we did not model blinking behaviour since this happens on a very short timescale of milliseconds. We assumed that every wavelength of light induces different switching rates, and that not all of the FPs are in the fluorescent state initially. We think that this led to the smallest possible model that describes bleaching, irreversible bleaching, and photochromicity.

We model the FPs as having three states, a natural (*nat*) state, a dark (*dark*) state, and an irreversible dark state (*irrdark*). In the natural state, the FPs are fluorescent: light is emitted after exposure to light in their defined absorption range. Both under light exposure and spontaneously, FPs can transition from the natural state to the dark state. In the dark state, the FPs are not fluorescent, but can return to the natural state both under the influence of light as spontaneously. For four different wavelengths, we include different rate constants for these transitions, as well as for the spontaneous transitions. FPs can also transition spontaneously from the dark state to an irreversible dark state. In this irreversible dark state no fluorescence is possible and during the course of the experiment, the FPs will not turn back to a fluorescent state anymore.

1.1 Equations

The transition rates between the states are defined as

$$\begin{aligned} v_{nat\to dark} = nat \cdot \Big(&k_{nd-spont}, \\ &+ k_{nd-RFP} \cdot P_{RFP}, \\ &+ k_{nd-GFP} \cdot P_{GFP}, \\ &+ k_{nd-CFP} \cdot P_{CFP}, \\ &+ k_{nd-YFP} \cdot P_{YFP}\Big). \\ v_{dark\to nat} = dark \cdot \Big(&k_{dn-spont} \cdot dark, \\ &+ k_{dn-RFP} \cdot P_{RFP}, \\ &+ k_{dn-GFP} \cdot P_{GFP}, \\ &+ k_{dn-CFP} \cdot P_{CFP}, \\ &+ k_{dn-YFP} \cdot P_{YFP}\Big). \\ v_{dark\to irrdark} = {}& dark \cdot k_{di-spont}. \end{aligned}$$

In these rate equations P_{RFP} denotes the light power in the red-wavelength range (which is around 570 nanometers), and similar for green ($\approx$ 490 nm), cyan ($\approx$ 438 nm) and yellow ($\approx$ 500 nm) light. The dynamics for the different FP-states are now determined by the following differential equations

$$\frac{\mathrm{d}nat}{\mathrm{d}t} = -v_{nat\to dark} + v_{dark\to nat},$$
$$\frac{\mathrm{d}dark}{\mathrm{d}t} = v_{nat\to dark} - v_{dark\to nat} - v_{dark\to irrdark}.$$

We assume there is no significant exposure to light before the experiment starts, and that the transition from dark to the irreversible dark state is negligible. We thus get an equilibrium between the natural and dark state, depending on the spontaneous rate constants. If we also rescale the state concentrations such that $nat(0) = 1$, we get

$$nat(0) = 1,$$
$$dark(0) = \frac{k_{nd-spont}}{k_{dn-spont}}.$$

1.2 Numerical solution and fitting procedure

To keep our approach as generic as possible, we decided to solve the differential equations numerically. In this way, the solutions can, in principle, also be found if the experimental protocol involves changing light powers. At constant light powers, the equations are analytically solvable, as we will show in Section 1.3.

Since the FPs can only be fluorescent when they are in the natural state, we fit the predicted dynamics of the natural state ($nat(t)$) to the fluorescence measurements. We fit all rate constants, so that we have 11 fitting parameters. In many experiments, however, the FPs will not be exposed to all wavelengths. The parameters, corresponding to the absent wavelengths, will then not be fitted.

Our set of experiments all involved a periodic exposure protocol, with short phases of light and longer phases of darkness. To make the fitting procedure computationally feasible, we approximated these experiments by calculating the average light power per wavelength.

1.3 Analytical solution

If the light powers at the different wavelengths are known, we can calculate overall rate constants

$$k_{nd} = k_{nd-spont} + k_{nd-RFP}\cdot P_{RFP} + k_{nd-GFP}\cdot P_{GFP} + k_{nd-CFP}\cdot P_{CFP} + k_{nd-YFP}\cdot P_{YFP},$$
$$k_{dn} = k_{dn-spont} + k_{dn-RFP}\cdot P_{RFP} + k_{dn-GFP}\cdot P_{GFP} + k_{dn-CFP}\cdot P_{CFP} + k_{dn-YFP}\cdot P_{YFP},$$
$$k_{di} = k_{di-spont}.$$

We are left with a set of linear differential equations,

$$\begin{bmatrix}\frac{\mathrm{d}nat}{\mathrm{d}t}\\ \frac{\mathrm{d}dark}{\mathrm{d}t}\end{bmatrix} = \begin{bmatrix}-k_{nd} & k_{dn}\\ k_{nd} & -k_{dn}-k_{di}\end{bmatrix}\cdot\begin{bmatrix}nat\\ dark\end{bmatrix}, \quad (1)$$

which can be solved by a sum of two exponentials. The integration constants can be calculated by demanding that $nat(0) = 1$, and $dark(0) = dark_0$. We further simplify the solutions by introducing

new variables:

$$\begin{aligned}\omega &= \sqrt{-4k_{di}k_{nd} + (k_{di} + k_{dn} + k_{nd})^2},\\ \alpha_+ &= k_{di} + k_{dn} + k_{nd} + \omega,\\ \alpha_- &= k_{di} + k_{dn} + k_{nd} - \omega.\end{aligned}$$

We are left with

$$\begin{aligned}nat(t) &= \frac{1}{2\omega}\left(e^{-\frac{1}{2}t\alpha_+}(-k_{di} - k_{dn} + k_{nd} + \omega - 2k_{dn}\,dark_0) + e^{-\frac{1}{2}t\alpha_-}(k_{di} + k_{dn} - k_{nd} + \omega + 2k_{dn}\,dark_0\right),\\ dark(t) &= \frac{1}{2\omega}\left(e^{-\frac{1}{2}t\alpha_+}(-2k_{nd} + (k_{di} + k_{dn} - k_{nd} + \omega)\,dark_0) + e^{-\frac{1}{2}t\alpha_-}(2k_{nd} + (-k_{di} - k_{dn} + k_{nd} + \omega)\,dark_0)\right),\end{aligned}$$

1.4 Biological interpretation of the parameters

A sum of two exponential decays

Note that both α_- and α_+ are positive, since

$$\omega = \sqrt{-4k_{di}k_{nd} + (k_{di} + k_{dn} + k_{nd})^2} < \sqrt{(k_{di} + k_{dn} + k_{nd})^2} = k_{di} + k_{dn} + k_{nd}.$$

Therefore, the solution is a sum of two contributions that decay exponentially in time. The exponential decay rates will differ by

$$\omega = \sqrt{-4k_{di}k_{nd} + (k_{di} + k_{dn} + k_{nd})^2} = \sqrt{(k_{nd} - k_{di})^2 + 2k_{dn}(k_{di} + k_{dn} + k_{nd})}.$$

If this difference is large, then the fluorescence dynamics will be bi-exponential, while if this difference is small, the decay could be fitted by a single exponential already. We see that bi-exponential behaviour will thus occur for example when k_{nd} is large compared to k_{di}. This is often the case.

The contribution that decays fastest, *i.e.* with the larger decay rate: α_+, corresponds to the FPs settling in a quasi-equilibrium between the dark state and the natural state. We call this a quasi-equilibrium because on the longer timescale, *i.e.* with rate α_-, fluorescence will decay by the transition of FPs from the dark to the irreversible dark state.

Approximating the change in fluorescence due to fast decay

The equilibration of natural and dark state can lead to both an increase or a decrease in fluorescence, depending on the initial conditions. The amplitude of this rapidly decaying contribution is given by the coefficient of $e^{-\frac{1}{2}t\alpha_+}$. Typically, the rate of the transition from dark to irreversible dark (k_{di}) will be smaller than the other rates, so that we can make the approximation $\omega \approx (k_{di} + k_{dn} + k_{nd})$. The change in fluorescence due to the fast decay is then approximated by

$$\begin{aligned}\text{change in fluorescence} &= -\frac{1}{2\omega}\left(-k_{di} - k_{dn}(1 + 2dark_0) + \omega - k_{nd}\right),\\ &\approx \frac{1}{2(k_{di} + k_{dn} + k_{nd})}\left(2k_{dn}\,dark_0 - 2k_{nd})\right),\\ &\approx k_{dn}\frac{\frac{k_{nd-spont}}{k_{dn-spont}} - \frac{k_{nd}}{k_{dn}}}{k_{dn} + k_{nd}}.\end{aligned}$$

If positive, this last quantity can be used as a measure of *FP photochromicity.* If light exposure decreases the ratio $\frac{k_{nd}}{k_{dn}}$ as compared to $\frac{k_{nd-spont}}{k_{dn-spont}}$, then fluorescence increases after exposure to light. This phenomenon is called photochromicity and is shown by several of the FPs that we have tested. We enlist these measures of photochromicity for all FPs in Table S1.

If negative, we do not speak about photochromicity, but the change in fluorescence will lead to a first fast decay of fluorescence, before the longer timescale, due to the lower rate constant k_{di} , will determine the dynamics.

Photochromicity can be induced by light of different wavelengths in various extents. Because we have measured fluorescence dynamics of the FPs under exposure to these different wavelengths, we can find out the photochromicity per wavelength if we reintroduce the wavelength-specific rate constants. This becomes a very complicated expression. However, if we linearly approximate the contributions of all wavelengths photochromicities (by calculating derivatives of the change in fluorescence with respect to the light powers P_{FP} at the point in which all powers are zero), then we find

$$\text{change in fluorescence-RFP} \approx k_{dn-RFP} \frac{\frac{k_{nd-spont}}{k_{dn-spont}} - \frac{k_{nd-RFP}}{k_{dn-RFP}}}{k_{dn-spont} + k_{nd-spont}} P_{RFP}. \tag{2}$$

The coefficient before P_{FP} is similar to the photochromicity measure and we take this as the photochromicity measure for light of this specific wavelength.

References

[Dean et al., 2011] Dean, K. M., Lubbeck, J. L., Binder, J. K., Schwall, L. R., Jimenez, R., and Palmer, A. E. (2011). Analysis of red-fluorescent proteins provides insight into dark-state conversion and photodegradation. *Biophys. J.*, 101(4):961–969.

[Dickson et al., 1997] Dickson, R. M., Cubitt, A. B., and Tsien, R. Y. (1997). On / off blinking and switching behaviour of single molecules of green fluorescent protein. 388(July):355–358.

[Morisaki and McNally, 2014] Morisaki, T. and McNally, J. G. (2014). Photoswitching-free FRAP analysis with a genetically encoded fluorescent tag. *PLoS One*, 9(9):1–9.

[Sinnecker et al., 2005] Sinnecker, D., Voigt, P., Hellwig, N., and Schaefer, M. (2005). Reversible photobleaching of enhanced green fluorescent proteins. *Biochemistry*, 44(18):7085–7094.

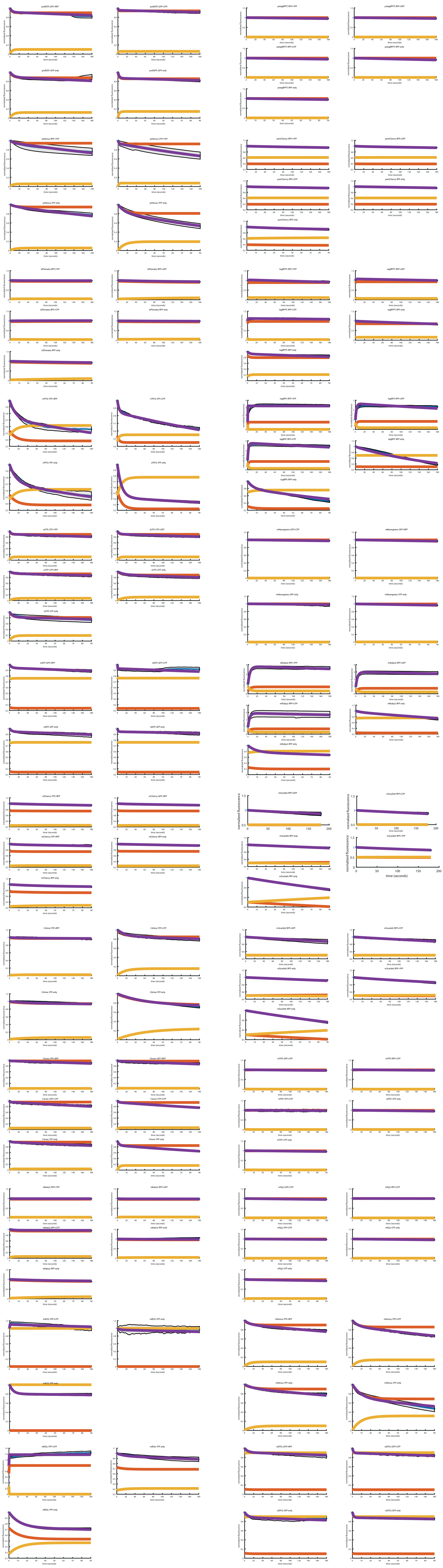

Figure S3. Photochromism and bleaching curves. Dots represent mean fluorescence values at the specific timepoint, normalized to the first frame. Shades indicate standard deviation. Red lines indicate the fitted natural (fluorescent) FP fraction. Yellow lines indicate the fitted reversible dark FP fraction. Blue lines indicates the fitted mean fluorescence, normalized to the first frame.
